# Towards a youth mental health paradigm: a perspective and roadmap

**DOI:** 10.1038/s41380-023-02202-z

**Published:** 2023-08-14

**Authors:** Peter J. Uhlhaas, Christopher G. Davey, Urvakhsh Meherwan Mehta, Jai Shah, John Torous, Nicholas B. Allen, Shelli Avenevoli, Tolulope Bella-Awusah, Andrew Chanen, Eric Y. H. Chen, Christoph U. Correll, Kim Q. Do, Helen L. Fisher, Sophia Frangou, Ian B. Hickie, Matcheri S. Keshavan, Kerstin Konrad, Francis S. Lee, Cindy H. Liu, Beatriz Luna, Patrick D. McGorry, Andreas Meyer-Lindenberg, Merete Nordentoft, Dost Öngür, George C. Patton, Tomáš Paus, Ulrich Reininghaus, Akira Sawa, Michael Schoenbaum, Gunter Schumann, Vinod H. Srihari, Ezra Susser, Swapna K. Verma, T. Wilson Woo, Lawrence H. Yang, Alison R. Yung, Stephen J. Wood

**Affiliations:** 1 Institute of Neuroscience and Psychology, University of Glasgow, Glasgow, UK; 2 Department of Child and Adolescent Psychiatry, Universitätsmedizin Berlin, Corporate Member of Freie Universität Berlin and Humboldt-Universität zu Berlin, Berlin, Germany; 3 Department of Psychiatry, The University of Melbourne, Carlton, VIC Australia; 4 Department of Psychiatry, National Institute of Mental Health and Neurosciences, Bangalore, India; 5 Department of Psychiatry, McGill University, Montreal, QC Canada; 6 Division of Digital Psychiatry and Department of Psychiatry, Beth Israel Deaconess Medical Center, Harvard Medical School, Boston, MA USA; 7 Department of Psychology, University of Oregon, Eugene, OR USA; 8 Office of the Director, National Institute of Mental Health, Bethesda, MD USA; 9 Department of Psychiatry, College of Medicine, University of Ibadan, Ibadan, Nigeria; 10 Orygen: National Centre of Excellence in Youth Mental Health, Parkville, VIC Australia; 11 Centre for Youth Mental Health, University of Melbourne, Parkville, VIC Australia; 12 Department of Psychiatry, University of Hong Kong, Hong Kong, China; 13 Departments of Psychiatry and Molecular Medicine, Donald and Barbara Zucker School of Medicine at Hostra/Northwell, Hempstead, NY USA; 14 Department of Psychiatry, The Zucker Hillside Hospital, Northwell Health, Glen Oaks, NY USA; 15 Centre for Psychiatric Neuroscience, Department of Psychiatry, Lausanne University Hospital, Lausanne, Switzerland; 16 Social, Genetic and Developmental Psychiatry Centre, Institute of Psychiatry, Psychology and Neuroscience, King’s College London, London, UK; 17 ESRC Centre for Society and Mental Health, King’s College London, London, UK; 18 Department of Psychiatry, The University of British Columbia, Vancouver, BC Canada; 19 Brain and Mind Centre, University of Sydney, Camperdown, NSW Australia; 20 Department of Psychiatry, Beth Israel Deaconess Medical Center and Harvard Medical School, Boston, MA USA; 21 Child Neuropsychology Section, Department of Child and Adolescent Psychiatry, RWTH, Aachen, Germany; 22 JARA-Brain Institute II, Molecular Neuroscience and Neuroimaging, Research Center Jülich, Jülich, Germany; 23 Department of Psychiatry, Weill Cornell Cornell Medicall College, New York, NY USA; 24 Departments of Pediatrics and Psychiatry, Brigham and Women’s Hospital/Harvard Medical School, Boston, MA USA; 25 Department of Psychiatry, University of Pittsburgh, Pittsburgh, PA USA; 26 Department of Psychiatry and Psychotherapy, Central Institute of Mental Health, Medical Faculty Mannheim/Heidelberg University, Mannheim, Germany; 27 CORE-Copenhagen Research Centre for Mental Health, Mental Health Center Copenhagen, University of Copenhagen, Faculty of Health and Medical Sciences, Department of Clinical Medicine, Hellerup, Denmark; 28 McLean Hospital/Harvard Medical School, Belmont, MA USA; 29 Centre for Adolescent Health, Murdoch Children’s Research Institute, University of Melbourne, Parkville, VIC Australia; 30 Departments of Psychiatry and Neuroscience, Faculty of Medicine and Centre Hospitalier Universitaire Sainte Justine, University of Montreal, Montreal, QC Canada; 31 Department of Psychology and Psychiatry, University of Toronto, Toronto, ON Canada; 32 Department of Public Mental Health, Central Institute of Mental Health, Medical Faculty Mannheim, Mannheim, Germany; 33 Centre for Epidemiology and Public Health, Health Service and Population Research Department, Institute of Psychiatry, Psychology and Neuroscience, King’s College London, London, UK; 34 The John Hopkins Schizophrenia Center, Johns Hopkins University, Baltimore, MD USA; 35 Division of Service and Intervention Research, National Institute of Mental Health, Bethesda, MD USA; 36 Centre for Population Neuroscience and Stratified Medicine, ISTBI, Fudan University, Shanghai, China; 37 Department of Psychiatry and Neuroscience, Universitätsmedizin Berlin, corporate member of Freie Universität Berlin and Humboldt-Universität zu Berlin, Berlin, Germany; 38 Department of Psychiatry, Yale University, New Haven, CT USA; 39 Program for Specialized Treatment Early in Psychosis (STEP), New Haven, VIC USA; 40 Departments of Epidemiology and Psychiatry, Columbia University, New York, NY USA; 41 New York State Psychiatric Institute, New York, NY USA; 42 Department of Psychosis, Institute of Mental Health, Buangkok, Singapore; 43 Duke-NUS Medical School, Singapore, Singapore; 44 Department of Psychiatry, Beth Israel Deaconess Medical Center, Boston, MA USA; 45 Laboratory for Cellular Neuropathology, McLean Hospital, Belmont, MA USA; 46 Department of Psychiatry, Harvard Medical School, Boston, MA USA; 47 Department of Social and Behavioral Sciences, New York University, New York, NY USA; 48 Department of Epidemiology, Columbia University, New York, NY USA; 49 School of Medicine, Faculty of Health, Deakin University, Melbourne, VIC Australia; 50 Department of Psychology and Mental Health, School of Health Sciences, Faculty of Biology, Medicine and Health, University of Manchester, Manchester, UK

**Keywords:** Psychiatric disorders, Diagnostic markers

## Abstract

Most mental disorders have a typical onset between 12 and 25 years of age, highlighting the importance of this period for the pathogenesis, diagnosis, and treatment of mental ill-health. This perspective addresses interactions between risk and protective factors and brain development as key pillars accounting for the emergence of psychopathology in youth. Moreover, we propose that novel approaches towards early diagnosis and interventions are required that reflect the evolution of emerging psychopathology, the importance of novel service models, and knowledge exchange between science and practitioners. Taken together, we propose a transformative early intervention paradigm for research and clinical care that could significantly enhance mental health in young people and initiate a shift towards the prevention of severe mental disorders.

## Rethinking Mental Health as “Youth Mental Health”

“Mental disorders are chronic diseases of the young.” [1]

Mental disorders constitute a major challenge to both society and science. Syndromes such as schizophrenia, depression, anxiety, and personality disorders comprise some of the largest disease burdens worldwide [2]. Despite the promises of genetics and translational neuroscience, insights into the causal mechanisms of major syndromes remain rudimentary [3], and the search for biomarkers to improve diagnosis and stratification has largely been unsuccessful [4]. Moreover, effect sizes for current pharmacological and psychological treatments are overall modest [5] and a significant number of patients will not respond to treatments [6].

**Fig. 1:**
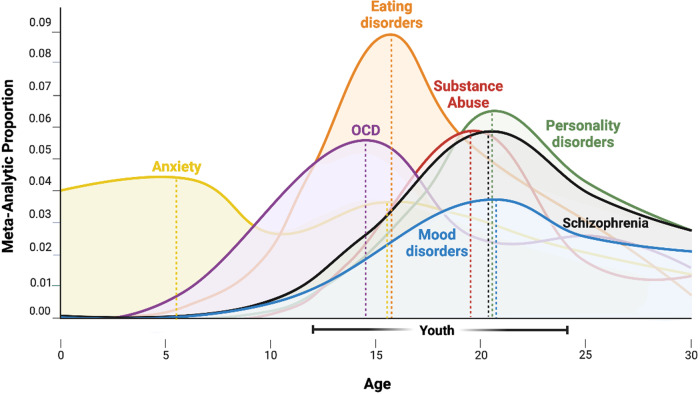
Age of onset of mental disorders. Distribution of age of onset of mental disorders in the general population based on the meta-analysis by Solmi et al. [9]: Meta-analytic epidemiologic proportion (y-axis) for anxiety disorders (5.5/15.5 years), substance use disorders (19.5 years), schizophrenia/psychotic disorders (20.5 years), eating disorders (15.5 years), personality disorders (20.5 years), obsessive-compulsive (14.5) and mood disorders (20.5 years) (ICD-10 blocks). The dotted horizontal lines represent the peak age of onset for each diagnostic category.

A cardinal feature of the existing paradigm in mental health has been its emphasis on fully established disorders in adulthood while early intervention and prevention have been relatively neglected [7, 8]. However, there is now consistent epidemiological evidence that has highlighted that all major syndromes constituting approximately 75% of mental disorders begin before the age of 25 years [9, 10] (Fig. 1).

In this perspective, we will make the case for a transformative paradigm in mental health that emphasizes early intervention and prevention of emerging mental disorders during youth, the period between 12 and 25 years of age^1^, with wide ranging implications for diagnosis, research, and interventions. Our approach is critically informed by the early intervention paradigm in psychosis [11]. Its scope has now been broadened to target emerging mental disorders during youth more generally given that young people with clinical high-risk criteria for psychosis (CHR-P) rarely present solely with signs of psychosis [12] and evidence that early identification and intervention is also potentially effective in personality disorders [13], eating disorders [14], and bipolar disorder [15].

The early intervention paradigm is furthermore motivated by the finding that young people face many barriers to accessing mental health care [16] during a developmental period, which is critical for social and occupational adjustment [17]. Importantly, the COVID pandemic has accelerated this trend with youth reporting a disproportionate increase in mental ill-health [18]. As a result, emerging mental disorders during youth frequently lead to sustained mental health problems during adulthood and lower overall functioning [17, 19].

To address these fundamental challenges, we set out core “pillars” for a youth mental health paradigm. Specifically, we propose that ongoing modifications in behavioral functions and underlying neural circuits suggest the presence of “sensitive periods” for the symptomatic expression of mental ill-health and corresponding “windows of opportunity” for early intervention. Secondly, risk factors interact with these sensitive periods on multiple levels that can be conceptualized as “developmental cascades”. Thirdly, novel diagnostic approaches are needed to facilitate interventions for sub-threshold symptomatic expressions of emerging psychopathology. Finally, the implications of these findings are discussed with respect to novel clinical and policy approaches and models for youth mental health.

## Brain development, sensitive periods, and emerging psychopathology

**Fig. 2:**
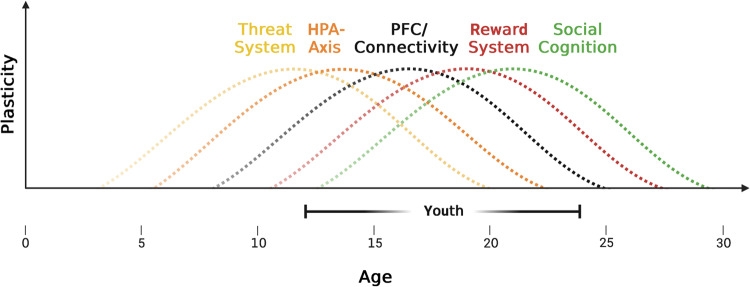
Sensitive periods during brain development. Overview of sensitive periods during brain development: The curves indicate the plastic potential for different neural systems between 0 and 30 years of age: (**a**) threat-regulation involving cortical-hippocampal-amygdala circuits (**b**) HPA-axis system (**c**) PFC/Connectivity subsumes local changes in PFC-properties (E/I-balance, Dopamine) as well as long-range connectivity with cortical-subcortical target regions (**d**) Reward System comprising striatum and connectivity with PFC and (**e**) social-Cognitive Processes. HPA-axis hypothalamic-pituitary-adrenal axis, PFC prefrontal cortex, E/I balance Excitation/Inhibition-balance, PFC prefrontal cortex.

An important principle in brain development is the notion of “sensitive periods” [20, 21]. Sensitive periods can be described as time-limited developmental windows during which environmental exposures have a pronounced effect on the functionality and organization of neural circuits and behavior as a result of heightened plasticity. Originally first described in the visual system [22], there is mounting data that several neural and cognitive systems that are relevant for emerging psychopathology, such as fear and stress regulation, higher cognitive processes, social cognition, and reward-processing, undergo time-limited modifications in circuit properties during youth (Fig. 2).

A core function that is impaired across many mental disorders is the ability to respond to threats and stress [23]. Importantly, anxiety disorders emerge during the first decade, peak around 15 years of age [9], and often persist into adulthood with significant effects on functioning and quality of life [24]. Recent work has identified unique changes in the neural circuits underlying threat regulation [25]. Specifically, fear extinction during adolescence is reduced in both humans and mice [26] and probing neural circuitry in mice has revealed altered synaptic plasticity and connectivity in prefrontal cortical-hippocampal-amygdala [26]. Interestingly in adolescent mice, if extinction training takes place in the threat-conditioning context that engages hippocampal-based circuits, extinction retention is significantly greater than that in the same-age counterparts that underwent extinction training in a novel context [27]. However, once outside this ‘sensitive period’, this form of context-based extinction has minimal additional effects.

The hypothalamic-pituitary-adrenal (HPA)-axis is the primary site for regulating the body’s stress response through releasing glucocorticoid hormones and HPA-axis dysregulation is involved in several mental disorders and contributes to emerging psychopathology [28]. Evidence from animal studies suggests that a variety of stressors are associated with elevated and prolonged HPA responses in youth compared with adulthood [29] and that differences in the psychopathological phenotypes observed may depend on the timing of the stress exposure during youth [30]. Conversely, environmental enrichment in juvenile animals can reverse the effects of prenatal stress and maternal separation [31, 32]. Together, these findings suggest that both risk and protective factors interact with HPA-functioning during youth [33].

In addition, ongoing modifications in higher-order cognitive functions, such as working memory (WM), response inhibition, and performance monitoring, as well as cognitive control are a central aspect of late brain development [34]. Anatomically, these functions are closely related to the integrity of prefrontal cortical (PFC) circuits, a region that is impaired in a range of mental disorders, including schizophrenia, depression, and bipolar disorder [35].

There is substantial evidence that the composition and interaction of the dopaminergic, glutamatergic, and GABAergic receptors in the PFC undergo profound changes during youth [34]. PV+ interneurons are of particular interest as they contribute towards the opening and closing of sensitive periods [21] and recent evidence suggests that PV+ interneuron expression continues to increase during youth while time-limited downregulation leads to permanent changes in E/I-balance during adulthood [36], a process which may be involved in emerging psychopathology [37].

Modifications of local PFC changes are accompanied by an extensive integration with cortical and subcortical areas through the maturation of long-range connections [38–42]. A core hypothesis underlying brain development is the view that ongoing changes in brain maturation lead to a period when limbic structures, such as the amygdala and striatum, predominate over prefrontal areas during youth [43, 44]. The change to predominance of executive prefrontal regions in adulthood reflect a potential neurobiological basis for improvements in emotion regulation which is critically impaired in affective disorders [45] but also in borderline personality disorders [46].

One important manifestation of the predominance of limbic processing over executive function is risk-taking behavior during youth [47], such as substance abuse. Evidence suggests that the likelihood of developing substance dependence is highest if substance abuse is initiated before age 14 [48], suggesting a sensitive period that is closely related to ongoing changes in incentive salience as well as changes in dopaminergic neurotransmission in the striatum and PFC [49] which is permanently altered by drug abuse [48]. In addition, tetrahydrocannabinol (THC), the principal psychoactive constituent of cannabis, may lead to a permanent disruption of E/I-balance and cognition during youth but not during adulthood [50].

Finally, converging findings have highlighted the possibility of a sensitive period for the development of the social-cognitive processes [51, 52]. Social cognition includes several domains, including theory of mind, emotion processing, and social cue identification, that have been linked to specific brain regions, such as the medial prefrontal cortex (mPFC) and tempo-parietal junction (TPJ) [53]. Recent evidence suggests that self-oriented thinking overlaps with regions required for understanding others, suggesting that the development of identity, a core task for youths, is an overlapping and intertwined process [52].

Reward circuits are particularly sensitive to peer influence during this age group, which can be related to higher levels of risk-taking behavior in social settings [54], and peer evaluations affect the self-image more than during other developmental periods [55]. Social interactions are also a necessary environmental exposure for establishing adult social behavior [56], a particularly pertinent issue given the widespread reduction of social interactions during the COVID-pandemic [57].

## Developmental cascades, the environment, and social-cultural context

Youth is a unique period during human development characterized by the onset of puberty, followed by psychosocial milestones, such as the separation from family, developing romantic attachments as well as discovering one’s sexual orientation and identity. The wide-ranging manifestations of youth across and within cultures suggest that this phase is highly shaped by the environmental context. Accordingly, understanding the relationship between sensitive periods, environmental factors, and the emergence of psychopathology necessitates the application of appropriate theoretical and conceptual frameworks that address the highly dynamic and context-dependent nature of this developmental period that in turn can be harnessed to identify risk and resilience factors for emerging mental ill-health.

The population neuroscience approach is well suited to applying a life-course epidemiology paradigm to mental disorders that acknowledges the complexity in time and space of environmental and genomic factors [58, 59]. In this context, it is important to understand and apply the concept of developmental cascades, which is integral to population neuroscience approaches in appreciating how transactions at different timescales (e.g., perinatal, infancy, adolescence, early adulthood), constructs (cognition, mood, behavior), and levels (molecular, physiology, individual, and social) have a domino effect on subsequent development [60]. These developmental cascades refer to the cumulative consequences of the many interactions and transactions occurring during development that result in spreading after-effects across levels, among domains at the same level, and across different systems [61].

Developmental cascades are potentially a useful approach to conceptualizing emerging psychopathology as many mental disorders in adulthood frequently have precursors in non-specific symptomatic manifestations during youth [62] but also in childhood [63, 64]. Recent evidence suggests that the risk of mental disorders is associated with elevated risk for other disorders and that younger age of onset is a predictor of longer duration of symptoms, comorbidity, and worse outcomes [19], highlighting the importance of interventions to target the earliest signs of mental ill-health.

Effects of specific environmental exposures on the outcomes of mental disorders and their behavioral precursors can be potentially mediated or moderated by a broad range of contextual, cultural, and biological factors. Mediators and moderators can, therefore, reveal time-sensitive windows for therapeutic interventions that target remediation of the exposure, mediating, or moderating factors. Thus, social media use has differential effects depending on the developmental timing [65], suggesting that sensitive periods during youth may be particularly malleable by environmental exposures [66].

Until recently, conceptualizing the multitude of environmental exposures and their contribution towards emerging psychopathology has been challenging. The exposome represents the totality of environmental exposures that an individual experiences from conception throughout the lifespan as well as the interaction among these exposures [67]. Choi et al. [68] examined genomic and exposome influences on internalizing and externalizing symptoms in youth, highlighting that additive and interactive influences of the genome and exposome explained over 30% and 60% of the variance in internalizing and externalizing symptoms while a single environmental risk factor accounted for only 1% of the variance.

## Emerging psychopathology and diagnostic framework(s)

The onset of the majority of mental disorders during youth [9] as well as the cascading and dynamic nature of psychopathology during development [19] have important implications for diagnostic frameworks. However, current diagnostic systems (DSM-5, ICD-11) have several shortcomings that require novel approaches to enable early detection and intervention. While several alternatives have been suggested, such as the Research Domain Criteria [69] and the Hierarchical Taxonomy of Psychopathology (HiTOP) [70], a developmental perspective on emerging psychopathology is rarely explicitly addressed (but see [71, 72]). In addition, both DSM-5 and ICD-11 do not allow a diagnosis to be made if symptom expression is below a certain cut-off value, even though there is established evidence for prodromal periods for psychosis [73] and bipolar disorder [74] as well as possibly for eating disorders [75], depression [76], and obsessive-compulsive disorders [77] (Table 1).

**Table 1:** Overview of early intervention for mental disorders in youth.

	Schizophrenia/ Psychotic disorders	Mood disorders (BP/MDD)	Personality disorders	Anxiety disorders	Substance abuse	OCD	Eating disorders
Target Population	CHR-P: [73]**** FEP: [97]****	BD: at-risk states*, prodrome (CHR-BP) [84]* MDD: Subthreshold* [135], Youth*** [136]	BPD subthreshold* Early-Stage BPD* [100]	At-Risk Populations [137]*** Youth [135]***	High-Risk Populations [101]*** Youth [138]***	Youth [77]**	Recent-Onset ED [102]**
Detection Screening Instruments (Sensitivity/Specificity, PPV, NPV) Duration of Prodrome (months) Duration of Untreated Illness (months) Relationship to Outcome	CHR-P: CAARMS, SIPS, SPI-A (67–100%, 39–100%, 24–100%) [139]** CHR-P: 21 [140]* FEP: 28–60 [94]*** Psychosis: 24-26 [141]*** FEP: Negative Symptoms, Self-harm [141]***	CHR-BP: BPSS-AS-P [142]* MDD: PHQ-9* [143] BP: 27 [74]*** MDD:1-36 [76]*** BP:60 [144]*** MDD:12-60 [145]*** BP: Onset of Mania, Psychosis [144]*** MDD: Treatment Response, Remission [145]***	BPD: MSI-BPD, BPQ, SCID-II BPD module (65-91) [146]* – – –	CMAS, MASC [147]* – GAD: 108* [148] Phobias: 120* [148] PTSD: 120* [148] –	CRAFFT (.80-86) [149]*** - Alcohol abuse: 108* [148] Alcohol Dependence: 72* [148] Drug Abuse: 72 [148] Drug Dependence: 30 [148]* -	SOCS (.67) [150]* - OCD: 84-120 [77]** OCD: Treatment Response [77]**	ED: SCOFF (100%, 87.5%) [151] EDE-Q (82.8%, 89.7%, 0.94) [152]* AN: 30 [95]** BN: 34 [95]** BED: 67 [95]** AN: Persistence of AN [153]*
Prognosis Assessment instruments (accuracy, AUC) Transition Risk Biomarkers Risk Calculators (AUC, C-Index):	CAARMS [154]***, SIPS [154]*** (0.85 pooled at 38 months)*** CHR-P: 17% at 1 year; 22% at 3 years) [73]*** CHR-P (Transition): Cognition [82]***, MRI [83]***, EEG [155]*** FEP (Functional Outcomes): [156]* CHR-P (Psychosis Risk): (0.70–0.80) [8]***	BPSS-FP (not available) SIBARS (0.7 at 18 months) [157]* 14% at 1 year [85]*; 23% at 2 years [85]* BP At-Risk (Transition): (.70) [158]* BP At-Risk (Onset of BD): (.71) [8]	– – – –	– – – –	– – – –	– – – –	– – – –
Interventions (level of evidence) Indicated Prevention Secondary Prevention	CHR-P: CBT (reduction in transition to psychosis) [98]**1b** FEP: Specialized EI- Services (psychosocial and pharmacological interventions: significant effects on functional and clinical outcomes) [97] **1b**	BP At-Risk: Pharmacology, Psycho-Social Interventions (no effect on transition, moderate effect on depression) [159]**1b** MDD: School-based interventions (small effect on symptoms) [160] MDD (Youth): Psycho-Social Interventions (no effect on onset but possible reduction of symptoms) **1b**	– Early-Stage BPD: EI Service Model (psychotherapy, befriending: greater treatment attendance/completion), [13]**1c**	School-Based Interventions: (small effect on symptoms) [161]**1b** Psychological/Educational Interventions: (small, preventive effect) [162]**1b** –	Cannabis: School-Based Interventions (small effect on cannabis use) [163]**1b** Youth with Substance Abuse: Motivational Interviewing (small effects on use) [164]**1b** Preliminary evidence for self-help/peer and CBT [101]**2b**	– –	Recent Onset ED: Specialized EI Service (improved clinical outcomes, reduction in admissions) [102]**2b**

* single study, ** systematic review, *** meta-analysis, **** umbrella review. Level of evidence:

1a) Systematic reviews (with homogeneity) of randomized controlled trials 1b) Individual randomized controlled trials (with narrow confidence interval) 1c) All or none randomized controlled trials 2) a Systematic reviews (with homogeneity) of cohort studies 2b) Individual cohort study or low quality randomized controlled trials (e.g. <80% follow-up) 2c) "Outcomes" Research; ecological studies 3a) Systematic review (with homogeneity) of case-control studies 3b) Individual case-control study 4) Case-series (and poor quality cohort and case-control studies) 5) Expert opinion without explicit critical appraisal, or based on physiology, bench research or "first principles"

*AN* Anorexia Nervosa, *APS* attenuated psychotic symptoms, *BED* Binge Eating Disorders, *BLIPS* brief limited intermittent psychotic symptoms, *BN* Bulimia Nervosa, *BP* Bipolar Disorders, *BPD* Borderline Personality Disorder, *BPSS-AS-P* Bipolar Prodrome Symptom Scale - Abbreviated Screen for Patients, *BPQ* Borderline Personality Questionnaire, *BPSS-AP* Bipolar Prodrome Symptom Interview and Abbreviated Screen for Patients, *BPSS-FP* Bipolar Prodrome Symptom Interview and Scale-Full Prospective, *CAARMS* Comprehensive Assessment of At Risk Mental States, *CHR-BP* Clinical High-Risk for Bipolar Disorder, *CHR-P* Clinical High-Risk for Psychosis, *CMAS* Children’s Manifest Anxiety Scale, *CBT* Cognitive Behavioral Therapy, *CRAFFT* Car, Relax, Alone, Forget, Friends, Trouble Questionnaire, *ED* Eating Disorders, *EDE-Q* Eating Disorders Examination Questionnaire, *EEG* Electroencephalography, *FEP* First Episode Psychosis, *GAD* Generalized Anxiety Disorder, *GRD* genetic risk and deterioration syndrome, *MASC* Multidimensional Anxiety Scale for Children, *MDD* Major Depressive Disorder, *MSI-BPD* McLean Screening Instrument for Borderline Personality Disorder, *MRI* Magnetic Resonance Imaging, *OCD* Obsessive Compulsive Disorder, *PHQ-9* Patient Health Questionnaire, *PTSD* Post-traumatic stress disorder, *SCID-BPD* BPD items from the Structured Clinical Interview for DSM-IV Axis II disorders (SCID-II) Personality Questionnaire, *SCOFF* Sick, Control, One, Fat, Food Questionnaire, *SIBARS* Semistructured Interview for Bipolar At Risk States, *SIPS* Structured Interview for Psychosis-Risk Syndromes, *SOCS* Self-report Short OCD Screener, *SPI-A* Schizophrenia Proneness Instrument, Adult version

CHR-P criteria were first developed for psychosis over 20 years ago [78] and comprise Attenuated Psychotic Symptoms (APS), Brief (and Limited) Intermittent Psychotic Symptoms (BLIPS or BIPS), and Genetic Risk and Deterioration Syndrome (GRD) (for details see [73]). CHR-P criteria are associated with high prognostic accuracy that is comparable to other paradigms of preventative medicine [79]. Thus, approximately 20% of individuals meeting CHR-P will develop a first episode of psychosis (FEP) in the initial 2 years [80]. There is evidence that clinical [81], cognitive [82], and neuroimaging measures [83] constitute possible biomarkers that significantly increase accuracy for predicting clinical outcomes in CHR-P participants.

Following the CHR-P paradigm, high-risk criteria were developed for bipolar disorders (CHR-BP) that comprise subthreshold mania and depressive symptoms [84]. Cross-sectional studies have indicated good internal reliability and consistency of these instruments [74]. First evidence suggests that CHR-BP criteria are associated with a conversion rate to bipolar disorder of 14.3% within 12 months [85].

**Fig. 3:**
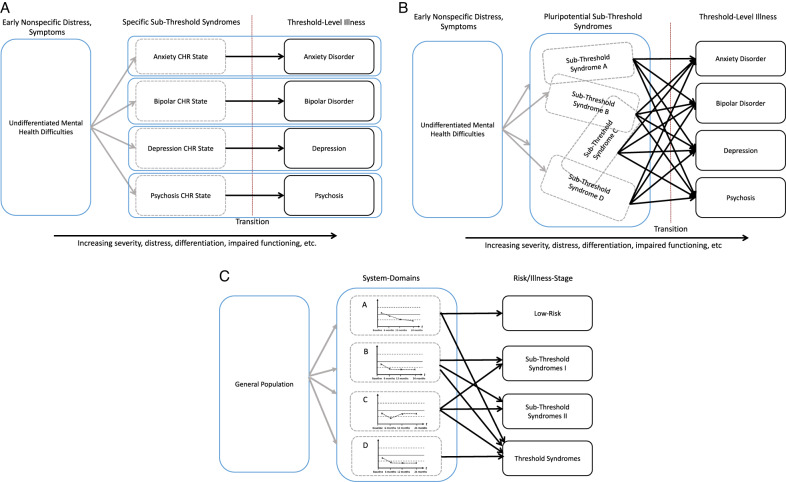
Diagnostic models in youth mental health. Diagnostic Models in Youth Mental Health: (**A**) diagnostic staging model focused on symptoms and functioning. **B** A transdiagnostic, pluripotential staging model in which variable subthreshold symptoms may overlap but give rise to a range of end-stage disorders. CHR indicates clinical high risk. **C** Growth Charts: Detection of emerging mental disorders in the general population. Four proposed domains of assessment and their age sex- and age-adjusted norms are displayed. Once a threshold of divergence from normative trajectories is reached, individuals could be offered options of closer tracking, more comprehensive assessments, or preventative or clinical interventions. The latter would range from low-risk preventive interventions when such departures begin to manifest clinically (at earlier stages), or treatment of manifest abnormalities that are functionally relevant and/or lead to distress (at later stages). Panels (**A**, **B**) are adapted from [165].

Building on the high-risk paradigm that is oriented along established diagnostic categories, alternative approaches have been advocated that reflect psychopathological dimensions [19, 86]. The *clinical staging framework* positions individuals along a *multidimensional gradient* of health to illness, capturing elements of risk, onset, course, and trajectory of illness [87, 88] (Fig. 3). Similar to clinical staging models in other areas of healthcare, staging frameworks hold the promise of guiding treatment selection, with less intensive interventions preferred at earlier stages and interventions with a higher risk/benefit ratio reserved for later stages [89].

Clinical staging models in youth mental health have been described in increasing levels of detail with some organized around specific diagnostic categories while others are transdiagnostic in nature [88]. The latter often aims to bridge the nonspecific, pluripotent nature of early-stage phenomena with more delineated presentations, extension to other dimensions of illness, and added layers of comorbidity seen in later stages. In integrating severity, multidimensionality, and pluripotentiality into a single model, clinical staging should be guided to develop a closer fit with youth-onset clinical syndromes with the ultimate goal of clinical utility [88].

Preventive approaches in the broader population may, however, require alternative approaches that are informed by data about normative development as well as vulnerability and risk exposures. Pediatric growth charts are a clinically valuable tool rooted in normative development and reflecting both physiology and external influences [90, 91]. Importantly, growth charts also allow the prediction of specific traits, such as height and weight, and if a deviation occurs then interventions to correct a possible developmental anomaly can be implemented during sensitive periods (Fig. 3).

A prerequisite for the application of growth charts in youth mental health is the availability of normative developmental data that comprise important domains of emerging psychopathology, such as cognition, emotion regulation, and sleep [92]. These can also be complemented by neuroimaging data, peripheral, digital, and genetic information as well as information about known risk factors and knowledge about sensitive periods. These multidimensional mental health growth charts would determine where an individual is located within normative development that in turn then lead to appropriate stage- and risk-adapted interventions.

## Developing and implementing tools for prevention and early intervention

The data on sensitive periods [23, 25, 33, 48, 49] as well as the peak incidence of mental disorders during youth [9, 10] have implications for service provision and treatments. Current approaches and service models, however, remain primarily organized around established diagnostic categories of mental disorders in adulthood [88] that impose further barriers for early intervention by the artificial distinction between child and adolescent as well as adult mental health services [93]. Accordingly, novel clinical approaches for youth mental health are required that emphasize early intervention, low-threshold service delivery, population-based prevention, novel technologies as well as translational and interactional research models.

### Sensitive periods and interventions for youth mental health

One important implication of sensitive periods during youth is that the timing of interventions is potentially critical for illness course and prognosis, suggesting that the plastic potential of neural circuits can be harnessed to modify ongoing developmental processes. Currently, however, substantial treatment delays for the majority of syndromes with an onset during youth, such as psychosis, bipolar disorders, and eating disorders, are pervasive [74, 94, 95] (Table 1), ranging from 2-5 years.

Duration of untreated psychosis (DUP) is an important determinant for symptomatic and functional treatment responses in FEP patients [94]. Similar data are available for BP [96], obsessive-compulsive [77] as well as eating disorders [95]. Moreover, there is evidence from indicated prevention in FEP-populations [97], suggesting that specialized psycho-social and pharmacological interventions can improve clinical outcomes compared to standard care [97]. In addition, there is evidence that CBT can reduce transition rates in CHR-P participants [98]. However, a more recent study suggested that clinical gains in FEP may not be sustained beyond the first two years of treatment [99].

Recent studies have extended the early intervention approach to personality disorders [100], substance abuse [101], and eating disorders [102]. Specialized psycho-social interventions for youth with borderline personality disorder (BPD) are effective in improving functioning and access to care [13] and early detection of eating disorders can improve prognosis, and decrease morbidity and mortality [14].

In order to maximize the benefits of the early intervention, we furthermore propose that behavioral and pharmacological interventions may benefit from incorporating data on sensitive periods [103]. Data from animal models of schizophrenia, for example, have shown that different behavioral and neurobiological interventions during the adolescent period but not during earlier or later developmental windows can completely rescue cognitive deficits and associated neural circuit dysfunctions [104–106].

The large majority of pharmacological and psycho-social interventions applied to youths have been developed for adult populations, potentially neglecting important differences in the properties of neural circuits as well as psychological variables. For example, a high proportion of youths with anxiety disorders do not respond to cognitive-behavioral therapy (CBT) [107] which may be due to inherent differences in fronto-amygdala circuitry [108]. Similarly, pharmacological interventions may also need to be adjusted given the ongoing modifications in neurotransmitter systems [34, 36].

A critical consideration in early pharmacologic intervention is minimizing side-effects. For many young people, early adverse experiences with psychotropic medication can reduce long-term adherence with all psychiatric treatments. As a result, the early intervention and the clinical staging models have encouraged investigations into more benign pharmacological treatments including “neutraceuticals”, that is, food or food products with health and medical benefits, such as fish oil [109], N-acetylcysteine [110], and cannabidiol [111], that may be suitable for targeting emerging psychopathology.

### Novel service models

The developmental timing of mental disorders and the unique social-cultural embedding of youth highlight the need for novel service models that also address the fact that young people have the most limited access to mental health services across the lifespan [16]. The first, and now most extensive example is the Australian *headspace model*, a national youth mental health service stream designed to provide highly accessible, youth-friendly centers that promote and support early intervention for mental and substance use disorders in young people [112, 113]. In addition to these face-to-face services, headspace also runs a 24/7 nationwide online support service (eheadspace; www.eheadspace.org.au). This service model has now been implemented in several countries [93].

A recent study investigated outcome data in 58.000 clients examining self-reported psychological distress, quality of life, and clinician-reported social and occupational functioning. The results showed that approximately 70% of young people who attended headspace centers in Australia significantly improved on at least one outcome measure [114]. However, functional improvements were observed in only 1/3 of cases which may reflect the fact that interventions were too short and not very intensive [115]. Headspace is a prominent example but not the only available model; further evaluations of youth mental health services are currently being conducted globally [116–119].

### Digital technologies

Early intervention approaches may also critically benefit from incorporating digital mental health technologies with unique opportunities and challenges [120]. However, Lettie et al. [121] highlighted that few studies using digital approaches and technologies targeting youth mental health have been replicated or assessed in real-world clinical settings. Many studies remain challenging to interpret given high rates of bias and few using causal methods to assess impact [122]. Furthermore, currently available commercial apps for youth mental health lack scientific evidence [123].

In contrast, there is emerging evidence for hybrid-interventions for youth mental health, involving both technology and some degree of human support. For example, the Moderated Online Social Therapy (MOST) offers intervention for youth diagnosed with early-stage psychosis, depression, help-seeking young people, and carers in a coherent platform [124–126]. New clinical models offering telehealth visits combined with a smartphone app, mindLAMP, involving both digital phenotyping for personalizing care and digital interventions for practicing skills, also show promise for rapidly reducing anxiety and depression-related symptoms [127]. Furthermore, a new generation of apps, such as EMIcompass, capture digital signals related to daily life (eg sleep patterns, mood) and use that data to respond with personalized and just-in-time support, thereby offering scalable and customized support for youth [128].

Digital technologies can also be used to detect emerging mental disorders outside established clinical pathways, an important prerequisite for population-based preventive approaches. In a recent study, a web-based screening platform allowed the identification of youth with CHR-P status as well as individuals with fully manifested psychosis with good sensitivity and specificity [129]. Digital phenotyping methods can also aid in relapse prediction by detecting changes in symptoms and behavioral patterns unique to each youth that may be associated with clinical deterioration [130]. Moreover, analysis of text messages on social media, in combination with machine and deep-learning techniques, may provide novel ways of identifying emerging mental disorders [131].

### Knowledge exchange between science and clinical care

The development of novel interventions for youth mental health requires consideration of the unique possibilities and requirements needed to enable the targeted search for preventive interventions and treatments. Specifically, we propose a bidirectional knowledge exchange that is reframed as a three-way interaction of democratizing research across researchers, clinicians, and youth with lived experience, including patients and carers [132], targeting the discovery of risk factors, mechanisms, and clinical responses to existing interventions in youth mental health [133]. Limiting youth and their families from the knowledge exchange network would be a critical oversight. Their participation in all aspects of youth mental health is thus vital to ensure that care provided is accessible, appropriate and effective. As such, co-design of interventions and services is an important aspect of youth mental health.

## Towards a paradigm for youth mental health

The converging findings from epidemiology, and basic and clinical research provide a powerful and complementary imperative for a “youth mental health paradigm” to guide science, practice, and policies. These are motivated by the highly plastic properties of neural circuits and associated cognitive and behavioral processes during youth which coincide with the peak incidence of major mental disorders between 12-25 years of age.

A critical implication of the timing of mental ill-health during youth and from the developmental cascade model is a broader focus on early manifestations of mental disorders as a primary focus for targeted interventions to prevent the occurrence of long-standing and chronic mental health conditions in adulthood [2]. In our view, this will require a shift towards early intervention models for a broad range of syndromes to enable selective and indicative prevention, low-threshold services for youth mental health [93], and population-based preventive approaches [8]. Here, prognostic algorithms that utilize knowledge of risk factors in combination with sensitive periods as well as biomarkers may be important for guiding clinical decision-making (Table 1).

The available evidence that early intervention is effective in psychosis [97, 134] as well as in other syndromes [8] together with the large unmet need in young people that has recently dramatically accelerated [9] provides additional impetus for such an endeavor. Several avenues towards interventions and implementation for youth mental health may follow from the framework outlined here that can be tested in large-scale studies. Firstly, prevention may focus on specific syndromes, such as the CHR-P paradigm vs. broader transdiagnostic phenotypes in youth mental health [88]. Secondly, population-based approaches that target the earliest manifestation of ill-mental health based on normative, developmental data vs. secondary prevention in clinical settings. Finally, the utility of knowledge derived from sensitive periods to guide the development and implementation of interventions in youth mental health remains to be demonstrated but could potentially be more effective for changing developmental trajectories vs. the application of established psycho-social and pharmacological therapies that were developed for adult populations.

While ambitious in scope, the benefits of such a youth mental health paradigm could be substantial and address the urgent need to improve the treatment of the most vulnerable age group for mental ill-health.

## Footnotes

1 We chose “youth” over other concepts, such as adolescence, as youth encompasses a broader definition of developmental phenomena and age-ranges.
